# Quality of life of older adults in Family Health Strategy: a cross-sectional study

**DOI:** 10.1590/1516-3180.2022.0445.R1.24042023

**Published:** 2023-07-17

**Authors:** Ilaise Brilhante Batista, Annah Thereza Mota, Ana Luiza Blanco, Jéssica da Silva Marinho, Maria Sortênia Alves Guimarães, Andréia Queiroz Ribeiro, Daniella Pires Nunes

**Affiliations:** I MSc. Nurse, Postgraduate Program in Health Science and Education, Universidade Federal do Tocantins (UFT), Palmas (TO), Brazil.; II BSc. Nutritionist, Department of Nutrition, Universidade Federal do Tocantins (UFT), Palmas (TO), Brazil.; III MSc. Gerontologist and Doctoral Student, Postgraduate Program in Gerontology, Universidade Estadual de Campinas (UNICAMP), Campinas (SP), Brazil.; IV MSc. Nurse, Postgraduate Program in Health Science and Education, Universidade Federal do Tocantins (UFT) Palmas (TO), Brazil.; V PhD. Nurse and Adjunct Professor Department of Medicine Course, Universidade Federal do Tocantins (UFT), Palmas (TO), Brazil.; VI PhD. Pharmaceuticals, Biochemistry and Adjunct Professor, Department of Nutrition and Health, Universidade Federal de Viçosa (UFV), Viçosa (MG), Brazil.; VII PhD. Nurse and Associate Professor, Medical-surgical Nursing Area, School of Nursing, Universidade Estadual de Campinas (UNICAMP), Campinas (SP), Brazil.

**Keywords:** Aged, Quality of life, Healthy aging, Health promotion, Family health strategy, Older adults, Cross-sectional study

## Abstract

**BACKGROUND:**

With the increase in the older adult population, it is essential to identify the living and health conditions that can impact the quality of life of these individuals.

**OBJECTIVES:**

To identify the domains and factors associated with the quality of life of older adults under the Family Health Strategy program.

**DESIGN AND SETTING:**

This was a cross-sectional analytical study was conducted in the municipality of Palmas, Tocantins, Brazil.

**METHODS:**

We assessed 449 older adults enrolled in the Family Health Strategy program. Data were collected between April and July, 2018. World Health Organization Quality of Life Assessment (WHOQOL-OLD) was used to assess the quality of life (QoL) and multiple linear regression was used to estimate the factors associated with QoL.

**RESULTS:**

The QoL domain with the highest score was death and dying (mean = 70.4), and the lowest score was for sensory functions (mean = 61.0 points). The factors associated with QoL were single marital status (β = -4.55; P = 0.014), level of independence for daily living activities (β = 4.92; P < 0.001), self-assessment of regular health (β = 5.35; P < 0.001), and poor health (β = -8.67; P < 0.001).

**CONCLUSION:**

The death and dying domain of QoL presented the highest score. Marital status, impairment in daily activities, and health self-assessment were associated with QoL.

## INTRODUCTION

Creating opportunities for healthy aging is becoming increasingly important as the global population continues to grow.^1^ Functional ability is a critical component of healthy aging, as it allows individuals to maintain their independence and quality of life as they age. Intrinsic capacity, which includes both physical and mental capacity, is an essential part of functional ability. Concordance between these attributes is a key determinant for the maintenance of quality of life and the factors that most negatively or positively influence this construct.^1,2^

Quality of life (QoL) is defined as “the individual’s perception of their position in life in the context of the culture and value systems in which they live and in relation to their goals, expectations, standards, and concerns”.^3^ In this way, QoL in old age is related to health as well as to physical, functional, cognitive, and emotional well-being, and other areas such as work, family, and daily living.^4^

The literature highlights conditions that are associated with good QoL among older people, including physical exercise, age, access to medical care, absence of depression, and fewer diseases.^5,6^ Factors that stood out in association with a worse QoL were cognitive decline and impairment of functional capacity and autonomy.^7,8^

In this context, primary healthcare is older adults’ access to health services through the Family Health Strategy (FHS) program, which continuously monitors users. Thus, it is important that the FHS multiprofessional and/or interdisciplinary team take a continuous and comprehensive look at the health of older adults and its impact on QoL.

Considering the increase in the older population, it is essential to identify the living and health conditions that can affect the QoL of older adults. These findings can assist FHS teams in carrying out actions to promote health and prevent health problems, as well as carry out interventions aimed at improving the QoL of the older population.

## OBJECTIVE

To identify the domains and factors associated with the quality of life of older adults under the Family Health Strategy program.

## METHODS

### Study design and participants

This cross-sectional and analytical study was carried out in Palmas, Tocantins, Brazil, from April to July, 2018. The study was conducted in accordance with the Strengthening of the Reporting of Observational Studies in Epidemiology (STROBE) guidelines.^9^

This study was approved by the Project and Research Evaluation Committee of Fundação Escola Saúde Pública de Palmas, Tocantins and the Human Research Ethics Committee of the Universidade Federal de Viçosa (CAAE: 84599718.5.0000.5153; date: April 09, 2018), opinion no. 2,587,419. All participants signed the Free and Informed Consent Term (FICT) after verbal and written explanations of the study.

The sample size was calculated considering the reference population of 9,978 older adults (n = 9,978),^10^ a prevalence of 50% (due to multiple outcomes of interest to the project, such as disability, hypertension, and others), tolerated error of 5%, design effect of 1, and a 95% confidence level.

The study population comprised 370 participants, with 20% added to cover possible losses, estimated at 449 individuals. Random sampling was used to select participants by drawing out those enrolled in FHS from a database previously organized in alphabetical order. A database was built using the names of the participants from the FHS records, which were organized by health unit and alphabetical order, for later drawing.

The eligibility criteria were as follows: aged 60 years or older, of both sexes, residents in the community, enrolled in the Family Health Strategy program of the municipality, and agreed to participate in the study by signing the informed consent form. The exclusion criteria were as follows: institutionalized or bedridden, upper and lower limb amputations, surgery on the arms or hands in the last three months, and impaired walking ability and requiring the aid of a cane or walker.

### Procedures

Data were collected from the Health Units of FHS through prescheduled interviews. Interviewers, trained by a team of professors and/or health professionals, administered a semi-structured, pretested questionnaire covering sociodemographic and health information, mostly consisting of precoded/closed-ended questions.

QoL, sociodemographic characteristics, and health conditions were evaluated using different assessment instruments.

*QoL* was assessed using the World Health Organization Quality of Life (WHOQOL-OLD), which consists of six domains (sensory functioning; autonomy; past, present, and future activities; social participation; death and dying; and intimacy). The final scores range from 0 to 100, with the highest score indicating the best QoL.

*Socioeconomic variables* included sex (male, female), age (60–69 years, 70–79 years, 80 years and older), marital status (married, single, divorced/separated, widowed), family arrangement (multipersonal and unipersonal), and education (1–4 years; over 4 years).

*Health conditions* included health self-assessment report (very good/good, regular, poor/very poor), multimorbidity (≥ 2 chronic diseases), polypharmacy (≥ 5 regular use of medications), cognition, performance in basic Activities of Daily Living (ADL) and Instrumental Activities of Daily Living (IADL), and history of hospitalization in the year prior to the interview.

*Cognitive impairment* was screened using the *Mini-Mental State Examination* (MMSE)^11,12^ with scores ranging from 0 to 30. A score of less than 20 was considered as cognitive impairment for individuals with no schooling, and less than 24 for individuals with schooling.^13^

The *Katz Index* was used to assess ADL (bathing, dressing, toileting, transferring, continence, and feeding)^14^ and older people who reported dependence on one or more activities were classified as dependent. IADL was measured using the *Lawton-Brody Scale*, which assesses a person’s ability to perform tasks such as using a telephone, shopping, preparing food, housekeeping, doing laundry, using transportation, handling medications, and handling finances.^15^ Older adults who scored between 26 and 27 were considered independent, and those who scored ≤ 25 points showed mild dependence.

### Data analysis

Data were entered into Microsoft Excel and analyzed using the Stata program, version 15.0, created by the Company Stata Corporation (College Station, United States). A descriptive analysis of the variables of interest was performed by estimating the absolute and relative frequencies and measures of central tendency and dispersion. The normality of the distribution of the quantitative variables was tested using the Shapiro-Wilk test. The means and respective 95% confidence intervals are presented for each domain of the QoL construct and the total score.

A multiple linear regression model was used to estimate the factors associated with QoL. Variables with P values less than or equal to 0.20 in the univariate analysis were selected for modeling. The variables that were adjusted or maintained association with QoL with P values less than or equal to 5% remained in multiple models.

## RESULTS

A total of 449 older adults participated in the study. Of the total sample, 50.5% were female. The mean age was 69.5 years (standard deviation, SD = 6.58 years), and most participants (57.7%) were between 60 and 69 years of age, married (60.5%), had one to four years of schooling (57.0%), and resided in multipersonal arrangements (85.2%). The occurrence rates of multimorbidity and polypharmacy were 60.8% and 23.2%, respectively. More than half of the seniors (53.4%) health self-assessed as regular, 82.4% were independent in performing ADL, and 65.9% were independent in performing IADLs.

The mean score for total QoL was 87.7 (95% confidence interval, CI = 86.16–87.99), with the highest mean score found for the death and dying domain (mean = 70.4; 95% CI = 68.10–72.90) and the lowest for sensory functioning (mean = 61.0 points; 95% CI = 59.90–62.20) (Table 1).

**Table 1:** Mean scores (95% CI) for the quality of life domains among older people. Palmas, Tocantins, Brazil, 2018 (n = 449)

Domains of the quality of life	Mean (95% CI)
Death and dying	70.4 (68.10–72.9)
Intimacy	67.8 (66.18–69.39)
Social participation	66.7 (65.28–68.09)
Sensory functioning	61.0 (59.90–62.20)
Autonomy	62.8 (61.27–64.25)
Past, present, and future activities	65.5 (64.08–66.87)
**Total**	87.7 (86.16–87.99)

CI = confidence interval.

The assessment showed that the highest score means of QoL were reported by men (mean = 88.72; 95% CI = 87.43–90.00), participants who reported being married (mean = 88 .15; 95% CI = 86.95–80.34), participants who rated their health as very good/good (mean = 92.30; 95% CI = 90.78–93.83), participants who reported having no multimorbidity (mean = 88.23; 95% CI = 86.68–89.79), and participants who reported being independent to perform IADL (mean = 89.30; 95% CI = 88.29–90.32) and ADL (mean = 87.82; 95% CI = 86.83–8.81) (Table 2).

**Table 2:** Quality of life according to the sociodemographic and health characteristics of older adults. Palmas, Tocantins, Brazil, 2018 (n = 449)

Variable				Quality of life			
				Mean	95% CI	β†	P value
**Sex**							
	Male			88.72	87.43–90.00	1.00	
	Female			85.47	84.18–86.75	- 3.24	< 0.001
**Age**							
	60 to 69 years			86.20	84.94–87.47	1.00	
	70 to 79 years			88.11	86.67–89.54	1.90	0.058
	80 years and older			88.64	85.47–91.80	2.44	0.151
**Marital status**							
	Married			88.15	86.95–80.34	1.00	
	Single			84.19	81.17–87.21	-4.12	0.050
	Divorced/separated			86.37	83.92–88.83	-1.84	0.190
	Widower			85.05	83.03–87.08	-3.21	0.012
**Living arrangement**							
	Multipersonal*			87.05	86.06–88.04	1.00	
	Unipersonal**			87.23	84.71–89.75	0.18	0.890
**Health self-assessment**							
	Very good/good			92.30	90.78–93.83	1.00	
	Regular			86.06	84.92–87.19	-6.51	< 0.001
	Poor/very poor			81.41	79.11–83.71	-11.35	< 0.001
**Cognitive decline**							0.080
	No			87.47	86.46–88.47	1.00	
	Yes			85.39	83.16–87.62	-2.07	
**Multimorbidity**							
	No			88.23	86.68–89.79	1.00	
	Yes			86.33	85.1–87.45	-1.90	0.046
**Independence in IADL** *							
	No			82.76	88.11–84.42	1.00	
	Yes			89.30	88.29–90.32	6.53	< 0.001
**Independence ADL** *							
	No			83.58	81.29–85.87	1.00	
	Yes			87.82	86.83–8.81	4.23	< 0.001
		Hospitalization in the last year					
			No	87.31	86.40–88.39		
			Yes	85.26	82.88–87.65	-2.13	0.102
**Polypharmacy**							
	No			87. 1	86.01–88.12	1.00	
	Yes			86.96	84.83–89.01	-0.14	0.895

IADL = instrumental activities of daily living; ADL = activities of daily living; CI = confidence interval.

* Multipersonal: living with more than one person (spouse, child, son/daughter-in-law, or grandchild)

** Unipersonal: living alone

† Univariate Linear Regress.

Table 3 shows the factors associated with QoL. Being single (β = -4.55; P = 0.014), independence in instrumental activities of daily living (β = 4.92; P < 0.001), and health self-assessment report as regular (β = -5.35; P < 0.001) and as poor/very poor (β = -8.67; P < 0.001) were associated with QoL.

**Table 3:** Final multiple regression model for factors associated with the quality of life of older adults. Palmas (TO), Brazil, 2018 (n = 449)

Variable		β*	95% CI	P value
**Marital status**				
	Married	1.00		
	Single	-4.55	-8.16; -0.94	0.014
	Divorced/separated	-1.20	-3.63; 1.23	0.331
	Widower	-0.95	-3.37; 1.46	0.438
**Health self-assessment**				
	Very good/good	1.00		
	Regular	-5.35	-7.30; -3.40	< 0.001
	Poor/very poor	-8.67	-11.34; -6.00	< 0.001
**Independence in IADL** (yes)		4.93	3.07; 6.77	< 0.001

CI = confidence interval; IADL = instrumental activity of daily living.

* Model adjusted for sex, age and multimorbidity.

## DISCUSSION

In the present study, the facet of QoL with the highest mean score was death and dying (mean = 70.4), indicating that the perception of death had no negative influence on participants’ QoL. This result is in agreement with other studies carried out with Brazilian older adults.^16–18^ According to Ermel et al.,^16^ older Brazilian individuals have a greater fear of feeling pain before dying than the fear of death itself. This finding may also be associated with psychological resilience among older adults.

Psychological resilience can be viewed as the set of personal resources that individuals acquire throughout their life and which help them to positively adapt to risks, threats, and losses over time.^19^ In other words, older people can be less concerned about death precisely because they are more resilient in the face of adversity, as they have experienced multiple losses of partners or spouses, family members, friends, and social roles, as well as declining health.^20^

In contrast to death and dying, the sensory functioning facet presented the lowest score for QoL in this study (mean = 61.0), followed by facet autonomy (mean = 62.7). This finding is in line with several studies since sensory loss becomes more common with aging, negatively impacting the QoL, functional capacity, and autonomy of older adults.^21,22^

A systematic review^22^ on sensory ability evaluated 23 studies to determine the relationship between QoL and hearing loss/impairment and found that limitations in activities of daily living and a decrease in social and emotional resources were the main causes of a lower QoL. These limitations affect the intrinsic capacity of an individual because they are associated with all the activities that older adults perform over time. In addition, their ability to communicate and socialize might be reduced, thereby worsening their isolation. The association between these factors predisposes individuals to a greater propensity to depression, sadness, and anger, resulting in a low QoL and the impairment of healthy aging.^1,22^

With increasing age, certain sensory losses affect the way older individuals experience the world and react to it, which may lead to difficulty in performing basic and instrumental activities of daily living, and a consequent increase in dependence. Attention is drawn to these changes as they can be detected early in FHS, contributing to extending the QoL and functionality of older adults.

In the population studied, the factors associated with QoL that stood out were being single, a health self-assessment of poor, and independence in instrumental activities of daily living.

Marital status is an important health determinant, and with advancing age, being married is a protective factor against depression and anxiety, and improves the QoL. Most married individuals, when compared to those who did not have a partner, presented a better assessment of QoL than single and widowed older adults.^23, 24^

The results of this study indicate that social and affective ties become more important with age. The lack of a social support network or inappropriate support is a predictor of mortality and is associated with higher rates of depression, disability, loneliness, and poor QoL in older people.^25^ However, a review showed that widowed and divorced individuals are more exposed to a greater risk of lack of support when compared to single individuals, because the latter tend to form more friendship ties over the years, which can act as a support network. ^25^

In relation to health self-assessment, a worsening in QoL was found among those self-rated as regular (β = -5.35) and as poor/very poor (β = -8.67). Health self-assessment reflects the knowledge and beliefs that individuals have about their health, considering physical, cognitive, and emotional indicators, and is a good predictor of morbidity, mortality, and functionality among older adults.^26^

A study on the health self-assessment of older participants showed a higher prevalence of self-ratings of poor/very poor and its association with aspects of physical and mental health, sense of happiness, and socioeconomic status.^27^ Other authors reported that sociodemographic factors, health status, and functional impairment led to negative self-ratings among older people.^28^

In this study sample, the facet of independence in IADLs showed an average increase of 4.92 units in the QoL score (β = 4.92) compared with individuals dependent on assistance to perform certain instrumental tasks. This result corroborates data from another study conducted in the state of São Paulo, in which older adults with worse QoL scores were 3.5 times more likely to have functional disability in IADL.^29^

Although the present study could not establish an association between QoL and sex or age, the literature indicates that women have worse mean disability scores than men, which was not found in the multiple analyses. Part of this difference may be due to the male-female health-survival paradox, which considers behavioral aspects and exposure throughout life.^30^

Men are affected by diseases that lead to death more quickly, while women live longer than men; however, they suffer more chronic diseases that lead to disabilities and tend to live longer with disabilities. Thus, although women live longer, this does not necessarily mean that they do so with a high QoL.^30–32^ Other explanations for the comparatively worse QoL of women are based on cultural and gender aspects. As women work a triple shift (care, family responsibilities, and career responsibilities), they tend to experience greater mental overload.^33^

Nevertheless, this study found that age had no significant association with QoL, corroborating with the results of a study conducted with Portuguese and Spanish older adults that showed no statistical difference between the groups, indicating that age did not have a great influence on QoL.^34^ On the other hand, Nguyen et al. reported that increasing age influenced the scores of QoL. Both men and women aged 80 years and over were more likely to have a lower QoL than younger older adults.^33^

The main limitation of this study is its cross-sectional design, which does not allow the establishment of a cause-and-effect relationship between the variables. Furthermore, individuals who were completely dependent were not included in the sample, and the results could not be extrapolated to the older population. The strengths of our study lie in the representative sampling, which contributes to the external validity of the results. Additionally, it focuses on the QoL of participants from a region that has rarely been studied in the literature, allowing us to infer comparisons of the demands and specificities of the older population in each region.

Finally, this study emphasizes the relevance of QoL assessment in ensuring that the FHS provides comprehensive healthcare to older citizens. QoL should guide the management of conducts, treatments, and policies for the population assisted by the FHS as a part of the multidimensional assessment of older adults enrolled in the territory.^2,35^

Multidimensional assessment is the main axis that structures the FHS care system for older individuals, allowing the identification of life habits and family, social, and economic contexts that may contribute to improving or worsening the QoL. It detects risk factors and warning signs that may represent risks of decline in functional capacity and optimizes resource allocation and care strategies.^35^

Therefore, it can be concluded that the monitoring of QoL is important throughout the Healthcare Network, with an emphasis on screening factors associated with QoL through multidimensional assessment, to guide the actions of FHS health professionals and public administrators. Educational campaigns, training of health professionals on how to meet the biopsychosocial needs of older adults, and the creation of public policies for the active participation of senior citizens in the society are measures that can impart benefits to the QoL of individuals and should be prioritized by the contributors involved in the FHS.

## CONCLUSIONS

The results showed that the factors associated with the worsening of QoL of older adults were being single and health self-assessment of regular or poor/very poor, while the best QoL was perceived among older individuals who were independent in performing instrumental activities of daily living. The mean QoL score of the surveyed group was the highest for the death and dying facet, demonstrating the best score for QoL. In contrast, the sensory functioning facet had the lowest mean and worst perception among older adults.

The information provided by this study can assist in devising strategies to help older adults maintain a good quality of life as well as guide primary care health professionals in meeting the demands and needs of this population, applying care plans, and developing health actions to aid healthy and active aging.
